# Photosynthetic photon flux density affects fruit biomass radiation-use efficiency of dwarf tomatoes under LED light at the reproductive growth stage

**DOI:** 10.3389/fpls.2023.1076423

**Published:** 2023-02-27

**Authors:** Xinglin Ke, Hideo Yoshida, Shoko Hikosaka, Eiji Goto

**Affiliations:** ^1^ Graduate School of Horticulture, Chiba University, Matsudo, Japan; ^2^ Plant Molecular Research Center, Chiba University, Chiba, Japan

**Keywords:** dry matter partitioning, fruit sink strength, fruit yield, indoor farming, Micro-Tom, plant factory, source strength, vertical farming

## Abstract

This study aimed to analyze the effects of photosynthetic photon flux density (PPFD) on fruit biomass radiation-use efficiency (FBRUE) of the dwarf tomato cultivar ‘Micro-Tom’ and to determine the suitable PPFD for enhancing the FBRUE under LED light at the reproductive growth stage. We performed four PPFD treatments under white LED light: 200, 300, 500, and 700 μmol m^−2^ s^−1^. The results demonstrated that a higher PPFD led to higher fresh and dry weights of the plants and lowered specific leaf areas. FBRUE and radiation-use efficiency (RUE) were the highest under 300 μmol m^−2^ s^−1^. FBRUE decreased by 37.7% because RUE decreased by 25% and the fraction of dry mass portioned to fruits decreased by 16.9% when PPFD increased from 300 to 700 μmol m^−2^ s^−1^. Higher PPFD (500 and 700 μmol m^−2^ s^−1^) led to lower RUE owing to lower light absorptance, photosynthetic quantum yield, and photosynthetic capacity of the leaves. High source strength and low fruit sink strength at the late reproductive growth stage led to a low fraction of dry mass portioned to fruits. In conclusion, 300 µmol m^−2^ s^−1^ PPFD is recommended for ‘Micro-Tom’ cultivation to improve the FBRUE at the reproductive growth stage.

## 1 Introduction

Small-sized and short-season (Sun et al., 2006) dwarf tomatoes have the potential to become commercial fruit vegetables cultivated in a plant factory with artificial light (PFAL), otherwise known as a vertical farm. They also have other advantages, such as low light requirements (Kato et al., 2011) and high planting density (Meissner et al., 1997), compared to general tomato varieties. However, fruit vegetables such as tomatoes have longer growth cycles and lower harvest indices than leafy vegetables such as lettuce. A lower harvest index indicates that more dry mass production is required for the same yield. Therefore, more energy and electricity are required in a PFAL to produce tomatoes with the same yield as leafy vegetables.

More than half of the electric power is used for lighting in a PFAL (Ohyama et al., 2002; Graamans et al., 2018). Therefore, a significant reduction in electricity costs can be achieved by improving light-use efficiency. Radiation-use efficiency (RUE) can be defined as the ratio of the dry biomass produced to the amount of photosynthetically active radiation (PAR) captured by the crop and is a classic and important parameter for measuring radiation utilization in crops (Williams et al., 1965; Shibles and Weber, 1966). Tomato plants are divided into two parts: edible (fruits) and inedible (roots, stems, and leaves). Fruit biomass radiation-use efficiency (FBRUE) can be defined as the ratio of the dry mass of a plant’s fruits to the number of photosynthetic photons captured by the plant (Wheeler et al., 2008; Li et al., 2019). It is an important index for the commercial production of tomatoes, indicating the distribution of photoassimilates in fruits. Additionally, FBRUE is a bridge linking photosynthesis and production output.

For general cultivars, the FBRUE of tomato was 0.3 g mol^−1^ in NASA’s Biomass Production Chamber (Wheeler et al., 2008), 0.2 g mol^−1^ in a closed plant production system (Goto, 2011), and 0.36 g mol^−1^ in the Permanent Astrobase Life-Support Artificial Closed Ecosystem (Li et al., 2019) when tomatoes were harvested. Therefore, there is still room for improvement in FBRUE. However, few studies have been conducted to improve the FBRUE of dwarf tomatoes in PFALs.

Moreover, photosynthetic photon flux density (PPFD) is an important environmental factor affecting RUE and dry matter distribution, further affecting FBRUE. At the vegetative growth stage, a higher PPFD led to lower RUE in a dwarf tomato cultivar ‘Micro-Tom’, from 300 to 700 µmol m^−2^ s^−1^ PPFD (Ke et al., 2021). In addition, PPFD influences the dry mass distribution of fruits. Yan et al. (2018) reported that the dry matter partitioning of tomato (cultivar, ‘Ruifen882’) fruits under supplementary artificial light (total daily PAR integral of 15.4 mol m^−2^) was higher than that without supplementary light (total daily PAR integral of 12.4 mol m^−2^). However, the effects of PPFD on biomass production and its distribution to plant organs are highly cultivar- and growth-stage-specific. Compared to cultivars with large fruits, Dueck et al. (2010) found that supplementary lighting had less effect on cherry tomatoes in commercial crop management. However, no study has reported the effects of PPFD on biomass production and its distribution to fruits in dwarf tomatoes.

In addition, plant biomass production and distribution are related to source strength and fruit sink strength, respectively (Heuvelink, 1996; Marcelis, 1996). However, no study has elucidated the effects of PPFD on the source and fruit sink strengths of dwarf tomatoes in PFALs. This study had two main objectives. The main one was to analyze the effect of PPFD on the FBRUE of dwarf tomatoes and to determine a suitable PPFD for enhancing FBRUE at the reproductive growth stage. The other was to identify the effects of PPFD on the source strength and fruit sink strength of dwarf tomatoes during the reproductive growth stage. We assumed that higher PPFD decreases FBRUE by decreasing RUE and/or dry matter partitioning of fruits affected by source strength and fruit sink strength. To test the hypothesis, FBRUE, RUE, dry matter partitioning of fruits, source strength, and fruit sink strength were calculated at different PPFDs.

## 2 Materials and methods

### 2.1 Plant material and growth condition

We used a dwarf tomato cultivar, ‘Micro-Tom’ (*Lycopersicon esculentum*), as the test material. Tomato seeds were sown in urethane sponges and kept under dark conditions for 3 days at 25°C. The plants were cultivated under white LED lamps (LDL40S-N19/21, Panasonic Corporation, Osaka, Japan) after germination at a PPFD of 200 μmol m^−2^ s^−1^ in a cultivation room at the Matsudo campus, Chiba University, Japan. The plants were cultivated in the cultivation room with a photoperiod of 16/8 h (day/night), air temperature of 25/20°C (day/night), 1,000 μmol mol^−1^ CO_2_ concentration, and relative humidity of 70%. A 1/2 OAT house A nutrient (OAT Agrio Co. Ltd., Tokyo, Japan) was used 10 days after germination for all plants. The electrical conductivity (EC) and pH of the nutrient solution were set at 1.3 dS m^−1^ and 6.3, respectively. The nutrient solution was renewed weekly.

According to our previous study (Ke et al., 2021), red and blue LED lamps (CIVILIGHT, DPT2RB120Q33 40 type, Showa Denko K.K., Tokyo, Japan; R:B = 9:1) were used for cultivation 24 days after sowing (DAS). In addition, the PPFD at the canopy top was set to 300 μmol m^−2^ s^−1^. As uniform seedlings bloomed, they were evenly transferred and placed on four polystyrene foam boards in four containers (18.6 L, L 600 mm × W 300 mm × H 141 mm, SANKO Co. Ltd., Tokyo, Japan) at 35 DAS. Each container was subjected to one of the four treatments with different PPFDs in a growth chamber equipped with white LED lamps (customized lamp, color temperature: 4000 K; Showa Denko K. K., Tokyo, Japan). The different light treatments were W200 (PPFD: 200 μmol m^−2^ s^−1^, daily light integral (DLI): 11.52 mol m^−2^ day^−1^), W300 (PPFD: 300 μmol m^−2^ s^−1^, DLI: 17.28 mol m^−2^ day^−1^), W500 (PPFD: 500 μmol m^−2^ s^−1^, DLI: 28.80 mol m^−2^ day^−1^), and W700 (PPFD: 700 μmol m^−2^ s^−1^, DLI: 40.32 mol m^−2^ day^−1^). A spectroradiometer (USR-45DA; USHIO Inc., Tokyo, Japan) was used to measure the spectral photon flux distributions of the LED lamps (**Supplementary Figure S1**). The environmental elements, except for the light condition, were the same as before transplanting. The pH and EC of the nutrient solution were set at 6.0 and 2.1 dS m^−1^, respectively. The seedlings were planted at a density of 238.1 plants m^−2^ during the reproductive growth stage. Axillary buds and side shoots were pruned after appearance.

At 36 DAS, plants in each light treatment (except W200) were separated into three groups: not pruned (44 plants), pruned to one fruit per plant (16 plants), or one fruit per truss (4 plants). ‘Micro-Tom’ is a determinate tomato with no new leaf on the main stem after the first truss. Therefore, plants with one fruit per truss were used to test whether the fruit grown in the plant pruned to one fruit per plant reflected potential growth. If there was no significant difference in fruit size/dry weight when fruit load was doubled or tripled (fruits of one-fruit plants vs. fruits of one-fruit per truss plants), then the fruit size/dry weight of one-fruit plants can be regarded as potential fruit growth. All plants, except those that did not receive fruit pruning, had their proximal fruits removed during anthesis.

### 2.2 Growth measurement

Three or four plants without fruit pruning in each treatment were destructively sampled for biomass measurements at 36, 43, 50, 57, 64, 71, and 82 DAS. Plant organs were dried for at least 72 h at 80°C in a ventilated oven. Fresh and dry weights of the plant organs (leaves, stems, fruits, and roots) were measured. Plant height was measured from the base of the main stem to the top using a ruler. The leaf area (LA, cm^2^) was measured using a leaf area meter (LI-3000C, Li-Cor Inc., Lincoln, NE, USA). Specific leaf area (SLA, cm^2^ g^−1^) was determined by dividing LA (cm^2^) by leaf dry weight (g). The number of fruits and anthesis dates for each fruit were recorded. The measurements of the growth parameters were performed with two replicates using six to seven plants.

### 2.3 Leaf optical properties

A spectrophotometer (V-750, JASCO Corporation, Tokyo, Japan) was used to measure the reflection and transmission spectra (Gausman and Allen, 1973; Saito et al., 2020) of the first leaf from the top of the main stem (fully expanded and unshaded leaf) at 82 DAS of the plant with an integrating sphere unit (ISV-922, JASCO Corporation, Tokyo, Japan). The measured light spectrum ranged from 400 to 700 nm. Three or four plants without fruit pruning were sampled per treatment. For each wavelength, the absorptance was calculated as 100% minus reflectance and transmittance.

### 2.4 Leaf photosynthetic light response determination

The response of photosynthetic rate (Pn) to PPFD was also determined on the first leaf from the top of the main stem using a portable photosynthesis measurement system (LI-6400XT, LI-COR Inc., Lincoln, NE, USA) equipped with a 6400-02B LED light source (90% red light with a peak at 665 nm and 10% blue light with a peak at 470 nm) in a leaf chamber at 43, 64, and 82 DAS. Initially, the leaves were clamped into a cuvette at 1000 μmol m^−2^ s^−1^ PPFD until stomatal conductance and Pn remained stable. A PPFD gradient of 2,000, 1,500, 1,000, 800, 500, 300, 200, 100, 50, and 0 μmol m^−2^ s^−1^ was applied to the leaf surface. A leaf temperature of 25 ± 1°C, relative humidity of 65–70%, and 1,000 µmol mol^−1^ CO_2_ concentration were set. A flow rate was set at 500 mol s^−1^ to allow air to flow through the system. Three plants without fruit pruning were measured for each treatment group. The photosynthetic quantum yield (*ϕ*, mmol CO_2_/mol photon) is the ratio of the net photosynthetic rate to PPFD on the leaf (Singsaas et al., 2001; Skillman, 2008). As a result of fitting light response curves to a nonrectangular hyperbolic function (Cannell and Thornley, 1998), the photosynthetic capacity was derived (maximum net photosynthetic rate (*P*_max_)).

### 2.5 Radiation-use efficiency

RUE (g mol^−1^) was defined as the ratio of the accumulated total dry weight (*W*, g) to the integrated PPFD (*I*_PPFD_, mol) received by a plant (Ke et al., 2021).

The *I*_PPFD_ (mol) until day *t*_1_ is calculated as follows:


(1) $$I_{\text{PPFD}}=T\times \sum_{t=0}^{t_{1}}\left[\text{PLA}(t)\times ({\text{PPFD}}_{T}-\text{PPFD}(t))](0<t\leq t_{1}\right)$$

Where *T* is the light period of 1 day, 5.76 × 10^4^ s (16 h × 3,600 s h^−1^), PLA(*t*) is the projected leaf area (m^2^) of the plant on day *t*, PPFD*_T_* (mol m^−2^ s^−1^) is the PPFD at the top of the canopy and was set as a specific constant for each treatment, and PPFD(*t*) (mol m^−2^ s^−1^) is the PPFD at the bottom of the canopy on day *t*.

To maintain PPFDs at the top of the canopies, a quantum sensor (LI-190, Lincoln, NE, USA) and GaAsp photodiodes (G1118, Hamamatsu Photonics K. K., Shizuoka, Japan) were used, and the PPFDs were maintained at 200, 300, 500, and 700 μmol m^−2^ s^−1^ in W200, W300, W500, and W700, respectively. Quantum sensors and GaAsp photodiodes were used to measure the PPFD of 29–51 evenly distributed points at 36, 37, 38, 40, 42, 45, 47, 49, 55, 58, 63, 65, 69, 72, 75, 78, and 81 DAS at the bottom of the canopy. The intercepted PPFD of the canopy was equal to the difference between the average PPFD at the top and bottom. The intercepted PPFD proportion was calculated by dividing the intercepted PPFD by the average PPFD at the canopy top. The intercepted PPFD proportion and PLA between two consecutive measured values increased linearly, and those on unmeasured days were estimated based on the measured values.

Free imaging software (LIA 32 ver. 0.378, Yamamoto) was used to determine the PLA from photos of the canopy (Furuyama et al., 2017) on the same days that PPFD measurements were taken.

The RUE and integrated PPFD received by the plant until 36 DAS were estimated as 1.36 g mol^−1^ and 0.6 mol, respectively, based on the data shown in our previous study (Ke et al., 2021). The PLA of the canopy, rather than the individual plant, was determined for each measurement. The fitted regression line slope to illustrate the relationship between total dry weight and *I*_PPFD_ was used to evaluate RUE during the entire reproductive growth stage.

### 2.6 Fruit biomass radiation-use efficiency

**Figure 1:**
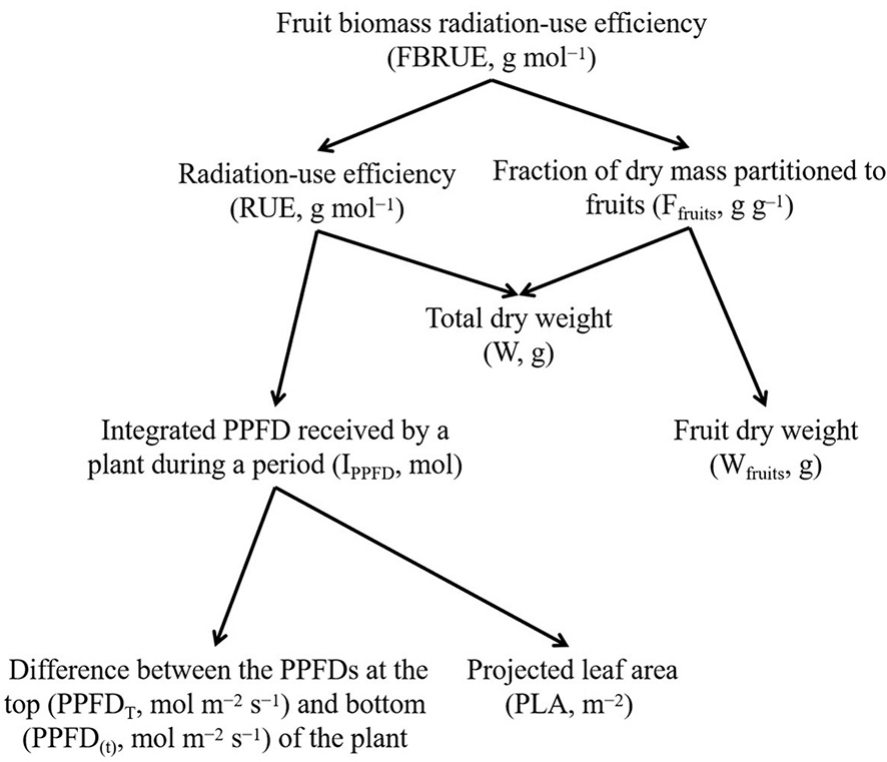
The scheme of fruit biomass radiation-use efficiency (FBRUE) segregated into underlying components. Arrows indicate the calculation of the parameters (i.e., lower-level components are required to calculate the parent parameters). Abbreviations and units for each component are indicated in parentheses.


It is possible to analyze the effects of PPFD on the FBRUE of a plant by breaking the effect down into its components (**Figure 1**). In this analysis, FBRUE is the product of RUE (g mol^−1^), and the fraction of dry mass partitioned into fruits (*F*_fruits_, g g^−1^) on a given day, as shown in the following formula:

(2) $$\text{FBRUE}=\text{RUE}\times F_{\text{fruits}}$$

*F*_fruits_ (g g^−1^) is defined as the ratio of the dry mass of tomato fruits to the total dry mass of the plant and is calculated using the following formula:


(3) $$F_{\text{fruits}}=\frac{W_{\text{fruits}}}{W}$$

Where *W*_fruits_ (g) is the fruit’s dry weight and *W* (g) is the dry weight of the whole plant on a given day.

Therefore, higher FBRUE can be caused by higher RUE and/or higher *F*_fruits_. An increase in RUE can be explained by an increase in *W* and/or a decrease in *I*_PPFD_. The latter is linked with a lower difference between the PPFDs at the top (PPFD*_T_*) and bottom (PPFD_(_ *_t_*_)_) and/or lower PLA. In addition, an increase in *F*_fruits_ is determined by a decrease in *W* and/or an increase in *W*_fruits_.

### 2.7 Source strength and fruit sink strength

Cumulative dry mass production of a ‘Micro-Tom’ plant from 36 to 84 DAS follows an exponential function in time according to a preliminary experiment and the present experiment (measured values shown in **Supplementary Figure S2** and the goodness of fit in the present experiment shown in **Supplementary Table S1**). Therefore, the total dry weight of a plant over time was calculated as follows:


(4) $$W(t)=\alpha \cdot e^{\beta t}$$

Where *W*(*t*) (g) is the total dry weight of the plant on *t* DAS and *α* and *β* are the coefficients based on the fitting function for the measured values.

The absolute growth rate is used as an estimate of the source strength (*S*_source_), which can be calculated as


(5) $$S_{\text{source}}(t)=\frac{dW(t)}{dt}$$

Where *S*_source_(*t*) (g day^−1^) is the rate of increase in total dry weight per plant on *t* DAS.

Fruit sink strength (*S*_fruit-sink_) is the sum of the sink strength of each fruit in a plant.

### 2.8 Sink strength of a single fruit

The sink strength of a single fruit can be quantified by calculating its potential growth rate (i.e., growth under nonlimiting assimilate supply conditions). In this study, nondestructive measurement of the hypothetical growth potential of fruits (i.e., one fruit per plant) was performed based on the method of Li et al. (2015).

The observation of fruit volume and age of plants with one fruit per plant was used to estimate the potential growth rate of a single fruit. The shape of the tomatoes was assumed to be an elliptical sphere. Therefore, the volume of tomato fruit was calculated as follows: fruit length × width × height × π/6. Measurements of the four OPF and four OPT plants were performed every 3 days.

The results demonstrated that the relationship between fruit volume and fresh weight of nonpruned fruits was almost the same as that of potential-growth fruits in ‘Micro-Tom’ (**Supplementary Figure S3**). To establish a linear regression between fruit volume and fresh weight, 77–104 randomly selected fruits were collected from the plants without fruit pruning in each light treatment.

Wubs et al. (2012) used a fourth-degree (or third-degree) polynomial function to express the relationship between fruit age and the dry matter content of individual fruits (IDMC_fruit_(*x*)).


(6) $${\text{IDMC}}_{\text{fruit}}(x)=ax^{4}+bx^{3}+cx^{2}+dx+e$$

Where *a*, *b*, *c*, *d*, and *e* are the coefficients and *x* is the fruit age (days after anthesis (DAA)). Preliminary experiments showed that pruning did not affect the relationship between fruit age and dry matter content in ‘Micro-Tom’ plants (data not shown). The dry weight of an individual fruit (IW_fruit_(*x*)) can be the product of IDMC_fruit_(*x*) and fresh fruit weight at *x* DAA.

Moreover, the Gompertz function can be used to fit the dry weight of individual fruits based on their age (Ji et al., 2020):


(7) $${\text{IW}}_{\text{fruit}}(x)={\text{IW}}_{\max}\times e^{-e^{-k(x-x_{m})}}$$

Where IW_max_ is the maximum dry weight of the fruit (g), *k* is the growth rate coefficient, and *x_m_* is the fruit age (DAA) at the maximum growth rate.

Based on the derivative of the Gompertz function, we obtained the growth rate of individual fruit (IGR_fruit_, g day^−1^) in relation to fruit age:


(8) $${\text{IGR}}_{\text{fruit}}(x)={\text{IW}}_{\text{fruit}}(x)\cdot k\cdot e^{-k(x-x_{m})}$$

Each fruit growth curve was fitted using a nonlinear mixed model, which assumed that measurements made on one fruit were grouped while assuming that the variation between measurements made on one fruit was lower than those made on different fruits.

### 2.9 Statistical analysis

One-way analysis of variance (ANOVA) was performed using SPSS for Windows (Version 24.0; SPSS Inc., Chicago, IL, USA) to analyze the data. A Tukey–Kramer test at *p* < 0.05 was used to compare the mean values of measured data to investigate significant differences among treatments.

## 3 Results

### 3.1 Growth characteristics

PPFD significantly affected the SLA, total fresh and dry weights, and total dry matter ratio (**Table 1**). SLA decreased with an increase in PPFD and was the lowest in W700. There were no significant differences in the total fresh and dry weights between W200 and W300. Total fresh and dry weights and dry matter ratio increased when PPFD increased from 300 to 700 µmol m^−2^ s^−1^. They were significantly higher under 700 µmol m^−2^ s^−1^ of PPFD than under 200 and 300 µmol m^−2^ s^−1^. However, PPFD had no significant effect on the plant height.

**Table 1:** Effect of photosynthetic photon flux density (PPFD) on the growth of ‘Micro-Tom’ 82 days after sowing (DAS).

Initial day or treatment	DAS	Plant height (cm)	Specific leaf area (cm^2^ g^−1^)	Total fresh weight (g)	Total dry weight (g)	Total dry matter ratio (%)
Initial day	36	9.9 ± 0.5	312.56 ± 17.88	10.00 ± 1.03	0.83 ± 0.09	8.40 ± 0.38
W200	82	13.1 ± 0.1	164.08 ± 2.28 a	109.40 ± 3.25 c	10.94 ± 0.23 c	9.68 ± 0.13 c
W300		11.1 ± 0.2	117.20 ± 2.68 b	101.78 ± 9.44 c	10.59 ± 0.97 c	10.43 ± 0.05 b
W500		11.0 ± 0.7	82.05 ± 2.18 c	129.21 ± 5.68 b	13.83 ± 0.63 b	10.70 ± 0.04 b
W700		11.5 ± 0.5	66.46 ± 3.19 d	160.07 ± 4.83 a	18.33 ± 0.51 a	11.46 ± 0.09 a

The initial day of the light treatment was 36 DAS. The growth parameters at 36 DAS are shown in the first row. Each value represents the mean ± standard error. Different letters in a column indicate significant differences among the treatments based on Tukey–Kramer’s test at *p* < 0.05 (n = 6−7). W200, W300, W500, and W700 denote 200, 300, 500, and 700 µmol m^−2^ s^−1^ PPFD treatments, respectively. All sampled plants are plants without pruning.

### 3.2 Leaf optical properties

The top leaves reflected more PAR in W500 and W700 than those in W200 and W300 (**Table 2**; **Supplementary Figure S4**). The maximum reflectance in W500 was 1.5% higher than the minimum ones in W200. The absorptance under red light decreased with an increase in PPFD from 200 to 500 µmol m^−2^ s^−1^ and was significantly higher in W200 and W300 than in W500 and W700. The absorptance of leaves in W200 was 1.1–2.3% higher than those in other treatments.

**Table 2:** Effects of PPFD on the reflectance, transmittance, and absorptance of leaves in the waveband of 400−700 nm in ‘Micro-Tom’ 82 DAS.

Treatment	Reflectance (%)	Transmittance (%)	Absorptance (%)
W200	5.7 ± 0.6 c	0.7 ± 0.1	93.5 ± 0.5 a
W300	6.2 ± 0.4 b	1.4 ± 0.4	92.4 ± 0.5 a
W500	7.2 ± 0.7 a	1.6 ± 0.4	91.2 ± 1.0 b
W700	7.1 ± 1.0 a	1.3 ± 0.2	91.6 ± 1.1 b

Each value represents the mean ± standard error. Different letters in a column indicate significant differences among the treatments based on Tukey–Kramer’s test at *p* < 0.05 (n = 4). All sampled plants are plants without fruit pruning.

### 3.3 Leaf photosynthetic light response determination

**Figure 2:**
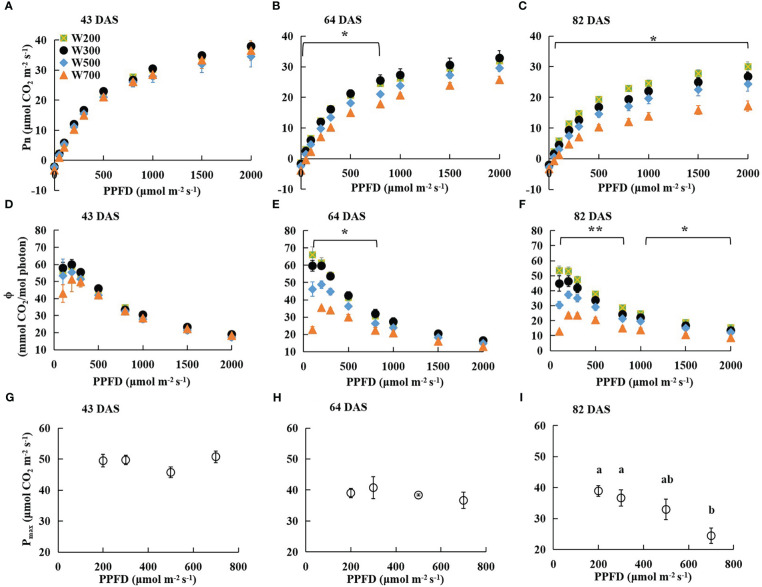
Effects of PPFD on light response curves of net leaf photosynthetic rate (Pn) 43 **(A)**, 64 **(B)**, and 82 **(C)** DAS, photosynthetic quantum yield (*ϕ*) 43 **(D)**, 64 **(E)**, and 82 **(F)** DAS, and photosynthetic capacity (maximum net photosynthetic rate (*P*_max_)) 43 **(G)**, 64 **(H)**, and 82 **(I)** DAS in ‘Micro-Tom’. Error bars show ± standard error. The asterisks in **(B, C, E, F)** indicate significant differences among treatments based on Tukey–Kramer’s test at ^*^ *p* < 0.05 and ^**^ *p* < 0.01 (*n* = 3−4). Different letters in **(I)** indicate significant differences among the treatments based on Tukey–Kramer’s test at *p* < 0.05. All sampled plants are plants without fruit pruning.

There were no significant differences in Pn measured at PPFDs from 0 to 2,000 µmol m^−2^ s^−1^ among all treatments at 43 DAS (**Figure 2A**). However, the Pn of leaves grown under higher PPFD was lower than that of leaves grown under lower PPFD at the same measured PPFD at 64 and 82 DAS (**Figures 2B, C**). At 64 DAS, the Pn measured at PPFDs ranging from 0 to 800 µmol m^−2^ s^−1^ in W200 and W300 was significantly higher than in W700 (**Figure 2B**). At 82 DAS, the Pn measured at PPFDs ranging from 50 to 2,000 µmol m^−2^ s^−1^ in W200 was significantly higher than in W700 (**Figure 2C**).

The *ϕ* in all treatments at 43 DAS (**Figure 2D**), in W300, W500, and W700 at 64 DAS (**Figure 2E**), and at 82 DAS (**Figure 2F**) increased as PPFD increased from 100 to 200 µmol m^−2^ s^−1^ and then decreased as PPFD increased to 2000 µmol m^−2^ s^−1^. There was no significant difference in the *ϕ* among all treatments at each PPFD at 43 DAS (**Figure 2D**). However, a higher PPFD led to a lower *ϕ* in W200 at 64 DAS (**Figure 2E**) and 82 DAS (**Figure 2F**). At 64 DAS, the values *ϕ* in W200 and W300 were significantly higher than those in W700 at PPFDs ranging from 100 to 800 µmol m^−2^ s^−1^ (**Figure 2E**). In addition, the *ϕ* in W200 was significantly higher than in W700 at PPFDs from 100 to 2,000 µmol m^−2^ s^−1^ at 82 DAS (**Figure 2F**).

The *P*_max_ of the first leaf was not significantly different among treatments at 43 DAS (**Figure 2G**) and 64 DAS (**Figure 2H**). *P*_max_ decreased with increasing PPFD at 82 DAS (**Figure 2I**). The *P*_max_ under 700 µmol m^−2^ s^−1^ PPFD was significantly lower than that under 200 and 300 µmol m^−2^ s^−1^ PPFD. However, there were no significant differences in Pn, *ϕ*, or *P*_max_ between W200 and W300.

### 3.4 RUE

**Figure 3:**
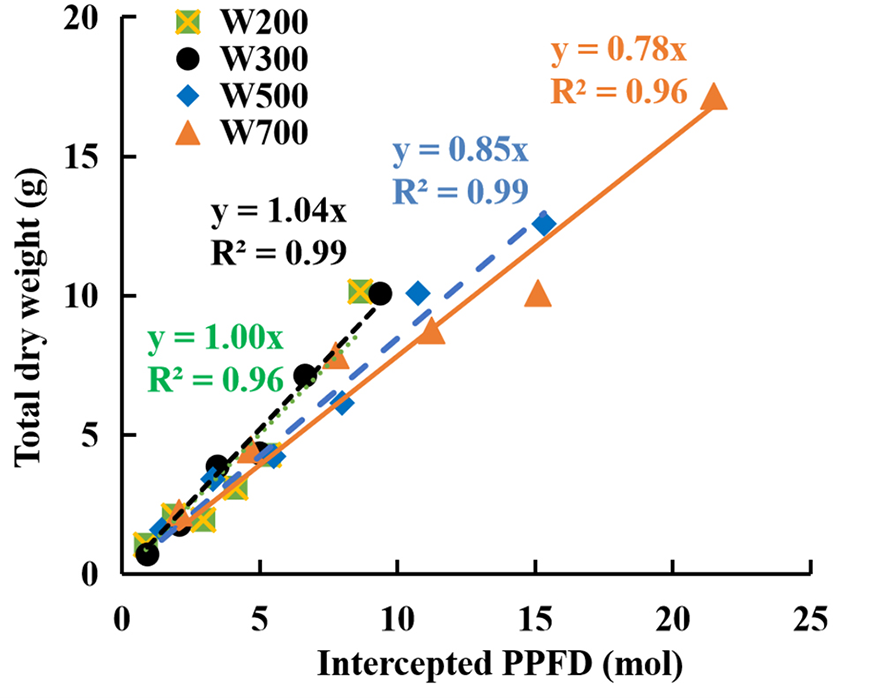
Relationships between accumulated total dry weights and cumulative intercepted PPFDs per plant in ‘Micro-Tom’ under different PPFDs during the reproductive growth stage. Each value represents the average of three or four plants without fruit pruning. The slope of the fitted linear relationship is the radiation-use efficiency (RUE, g mol^−1^) at the reproductive growth stage.

The fitted line slope in **Figure 3** indicates RUE during the reproductive growth stage. RUE increased marginally when PPFD increased from 200 to 300 µmol m^−2^ s^−1^ and then decreased with an increase in PPFD from 300 to 700 µmol m^−2^ s^−1^. The RUE was the highest (1.04 g mol^−1^) in W300 and the lowest (0.78 g mol^−1^) in W700 among the four treatments.

### 3.5 FBRUE component analysis and dry mass partitioning to fruits

**Figure 4:**
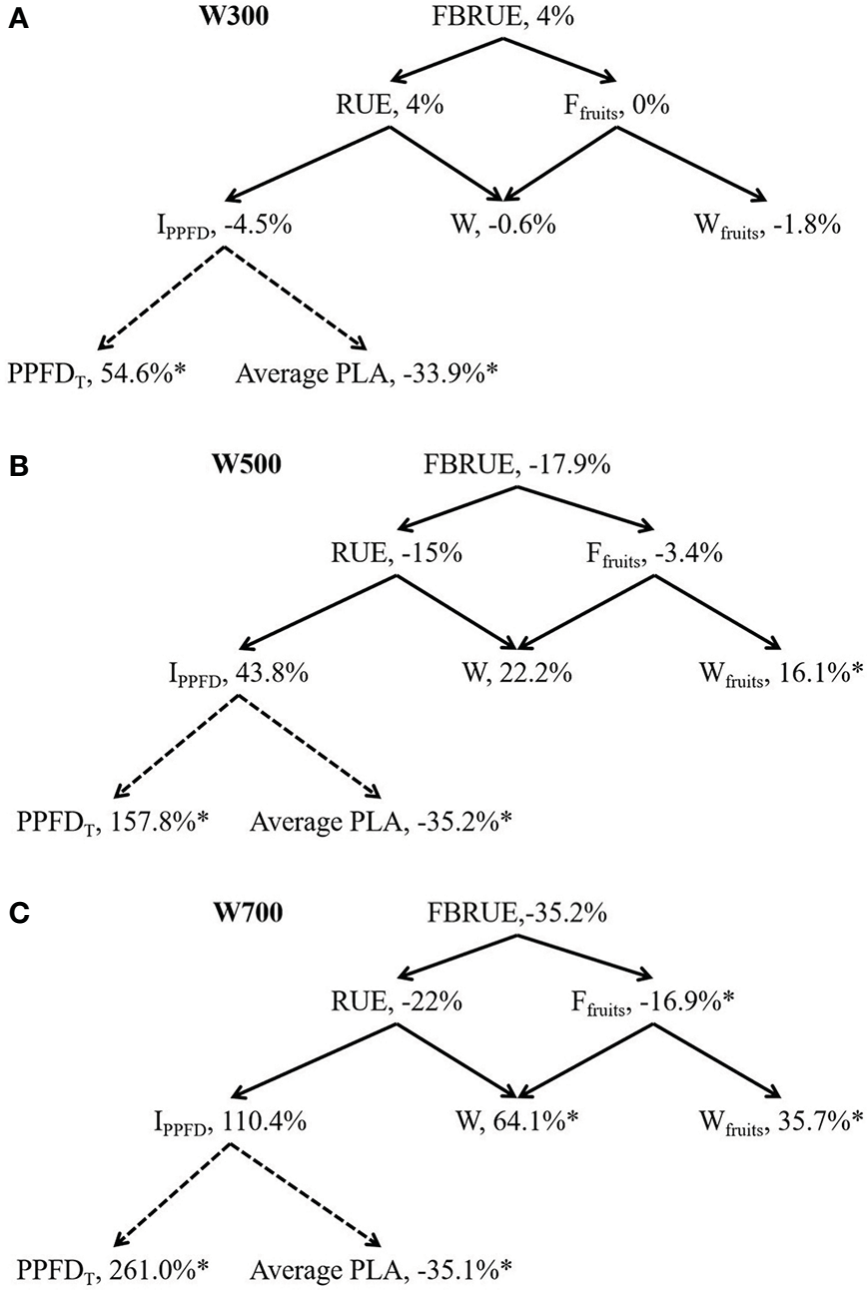
Fruit biomass radiation-use efficiency (FBRUE) component analyses under 300 **(A)**, 500 **(B)**, and 700 **(C)** µmol m^−2^ s^−1^ PPFDs at 82 DAS. The asterisks indicate significant differences among treatments based on Tukey–Kramer’s test at ^*^ *p* < 0.05 (*n* = 3−4). For black solid arrows, the arrowhead component is used to calculate the parent parameter in the tail. For black dotted arrows, the arrowhead component is affected by the tail component. Percentages are the increment relative to W200; all values in W200 are considered 100%. Abbreviations within schemes are as follows: FBRUE, fruit biomass radiation-use efficiency (g mol^−1^); RUE, radiation-use efficiency (g mol^−1^); *F*_fruits_, fraction of dry mass partitioned to fruits (g g^−1^); *I*_PPFD_, integrated PPFD received by the plant until 82 DAS (mol); *W*, total dry weight (g); *W*_fruits_, fruit dry weight (g); PPFD_T_, difference between the PPFDs at the top and bottom of the plant (mol m^−2^ s^−1^); average PLA, average projected leaf area (m^2^). All sampled plants are plants without fruit pruning.

FBRUE component analyses under different PPFDs are shown in **Figure 4** based on **Figure 1** to quantify the effects of PPFD on-increment or decrement of main factors of FBRUE. FBRUE, RUE, and *F*_fruits_ decreased with the increase in PPFD from 300 to 700 µmol m^−2^ s^−1^ (**Figure 4**). The FBRUE and RUE under 300 µmol m^−2^ s^−1^ PPFD were the highest. The *I*_PPFD_ until 82 DAS, *W*, *W*_fruits_, and PPFD*_T_* increased with an increase in PPFD from 300 to 700 µmol m^−2^ s^−1^. PPFD significantly affected *F*_fruits_, *W*, *W*_fruits_, PPFD*_T_*, and average PLA (**Supplementary Table S2**). Higher PPFD led to lower FBRUE because of lower RUE and *F*_fruits_ from 300 to 700 µmol m^−2^ s^−1^ (**Figure 4**). The decrease in RUE was greater than in *F*_fruits_ in the three treatments. The reason for the decrease in RUE with an increase in PPFD was that the increase in *W* was less than the increase in *I*_PPFD_.

**Figure 4A** shows that the difference in FBRUE between W200 and W300 was small because of the small differences in RUE and *F*_fruits_ between W200 and W300. The average PLA decreased by 33.9%, and there was a 4.5% decrease in the *I*_PPFD_ when the PPFD at the top of the canopy increased from 200 to 300 µmol m^−2^ s^−1^. The PPFD*_T_* and average PLA in W300 were significantly higher and lower than in W200. PPFD*_T_* and *W*_fruits_ in W500 were significantly higher than those in W200 (**Figure 4B**). The average PLA in W500 was significantly lower than that in W200. PPFD*_T_* and *W*_fruits_ in W700 were significantly higher than in W200 (**Figure 4C**). The *F*_fruits_ and average PLA in W700 were significantly lower than in W200.

**Figure 5:**
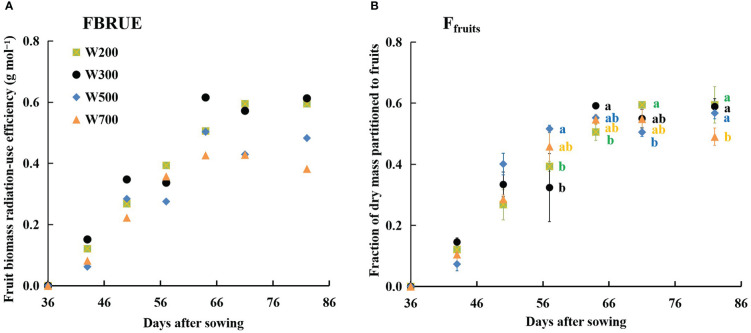
Effects of PPFD on fruit biomass radiation-use efficiency (FBRUE) **(A)** and the fraction of dry mass portioned to fruits (*F*_fruits_) **(B)** over time in ‘Micro-Tom’. All sampled plants are plants without fruit pruning. Different letters indicate significant differences among the treatments based on Tukey–Kramer’s test at *p* < 0.05 (*n* = 3−4) in **(B)**.

FBRUE increased rapidly and then flattened in all treatments (**Figure 5A**). The FBRUE increased slightly as the PPFD increased from 200 to 300 µmol m^−2^ s^−1^, decreased as the PPFD increased from 300 to 700 µmol m^−2^ s^−1^, and was the highest in W300 at 82 DAS.

The *F*_fruits_ increased from 36 to 64 DAS in all treatments and remained stable from 0.49 to 0.60 until 82 DAS (**Figure 5B**), showing the same trend with FBRUE (**Figure 5A**). At 57 DAS, the values of *F*_fruits_ in W200 and W300 were significantly lower than those in W500. The *F*_fruits_ was lowest under 700 µmol m^−2^ s^−1^ PPFD at 82 DAS. In addition, the *F*_fruits_ were the largest, and the fraction of dry mass partitioned to stems was the lowest among all organ fractions in all treatments at 50 DAS (**Supplementary Figure S5**).

### 3.6 The number of fruits and yield

The number of fruits and their fresh and dry weights increased with an increase in PPFD (**Table 3**). In W700, they were significantly higher than those in the other three treatments at 82 DAS. The number of fruits in W300 and W500 was significantly higher than in W200. The fresh and dry weights of the fruits in W200 and W300 were significantly lower than those in W500 and W700.

**Table 3:** Effects of PPFD on the number of fruits, fruit fresh and dry weight, and fruit dry matter ratio in ‘Micro-Tom’ 82 DAS.

Treatment	Number of fruits	Fruit fresh weight (yield, g)	Fruit dry weight (g)	Fruit dry matter ratio (%)
W200	10.6 ± 1.2 c	67.73 ± 6.54 c	6.53 ± 0.70 c	9.33 ± 0.33
W300	14.3 ± 0.6 b	66.76 ± 10.44 c	6.41 ± 1.05 c	9.75 ± 0.25
W500	15.0 ± 1.4 b	80.33 ± 4.88 b	7.58 ± 0.56 b	9.25 ± 0.25
W700	17.4 ± 1.6 a	90.70 ± 7.20 a	8.86 ± 0.83 a	9.75 ± 0.25

Each value represents the mean ± standard error. Different letters indicate significant differences at the *p* < 0.05 level among PPFD treatments with Tukey–Kramer’s test. Each value of the number of fruits, fruit fresh and dry weight, and fruit dry matter ratio represents a mean of six or seven values.

### 3.7 Source strength and fruit sink strength

**Figure 6:**
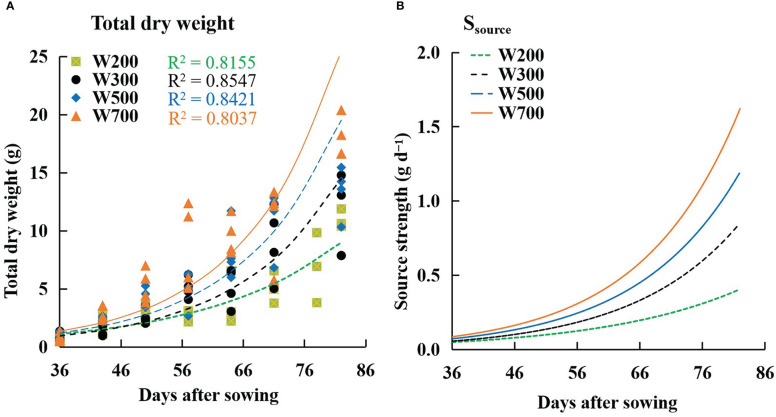
Effects of PPFD on the total dry weight of a plant **(A)** and source strength (*S*_source_) **(B)** over time in ‘Micro-Tom’. Symbols represent measured total dry weights in W200 (square), W300 (circle), W500 (diamond), and W700 (triangle). Curves represent exponential functions fitted for W200 (green), W300 (black), W500 (blue), and W700 (orange). *R* ^2^ is the coefficient of determination in W200 (green), W300 (black), W500 (blue), and W700 (orange), respectively. All sampled plants are plants without fruit pruning.

The *W* increased with time and PPFD (**Figure 6A**). The fitted curves followed an exponential function, and the *R* ^2^ values for all treatments exceeded 0.8. The same trend as *W* was observed in the *S*_source_ (**Figure 6B**). *S*_source_ was lowest under 200 µmol m^−2^ s^−1^ PPFD and highest under 700 µmol m^−2^ s^−1^ PPFD at all times among all treatments.

**Figure 7:**
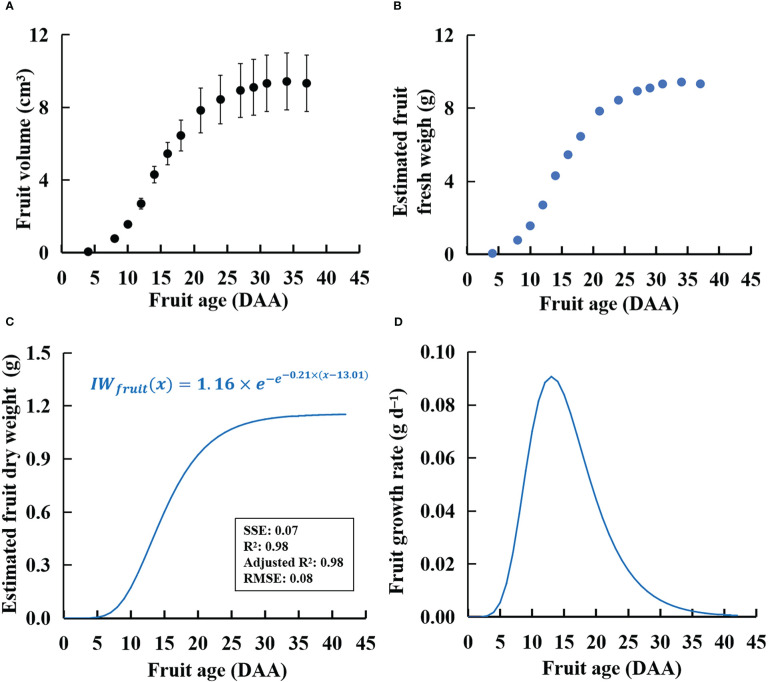
Calculated volumes (**A**, by measuring fruit diameters and heights in one-fruit plants), estimated fresh weight **(B)**, estimated dry weight **(C)**, and fruit growth rate **(D)** of an individual fruit with potential growth over time in ‘Micro-Tom’. The sample size in **(A)** was 12. The estimated dry weight **(C)** is IW_fruit_(*x*) in Eq. (7). The fruit growth rate in **(D)** is IGR_fruit_(*x*) in Eq. (8).

There were no significant differences in fruit volume and single fresh and dry weights between one-fruit plants and one-fruit per truss plants in W300, W500, and W700 (**Supplementary Table S3**). The relationships between fresh fruit weight and fruit volume of one-fruit plants were well fitted with linear regression without intercept (*R* ^2^ > 0.97 for all fits, shown in **Supplementary Figure S3**) in the three treatments. Moreover, the ratio of fresh weight to fruit volume of plants without fruit pruning was similar to that of the one-fruit plants (**Supplementary Figure S3**). As a result, this study assigned the ratio of fresh weight to fruit volume to 1.0 g cm^−3^. Specifically, PPFD had little effect on the ratio of fresh weight to fruit volume in ‘Micro-Tom’. There was no significant difference in fruit volume among the three treatments (**Figure 7A**). In addition, the fresh weight of the potentially growing fruits (**Figure 7B**) was estimated using the calculated fruit volume (**Figure 7A**).

There was no significant difference in fruit dry matter content among W300, W500, and W700 plants (data not shown). Changes in fruit dry matter content with time among the three PPFD treatments were slight during 9–42 DAA (**Supplementary Figure S6**). The IW_fruit_ in Eqs. (7) and (8) was 1.16 g (**Figure 7C**). The *k* and *x_m_* were 0.21 and 13 DAA in Eq. (7).

**Figure 8:**
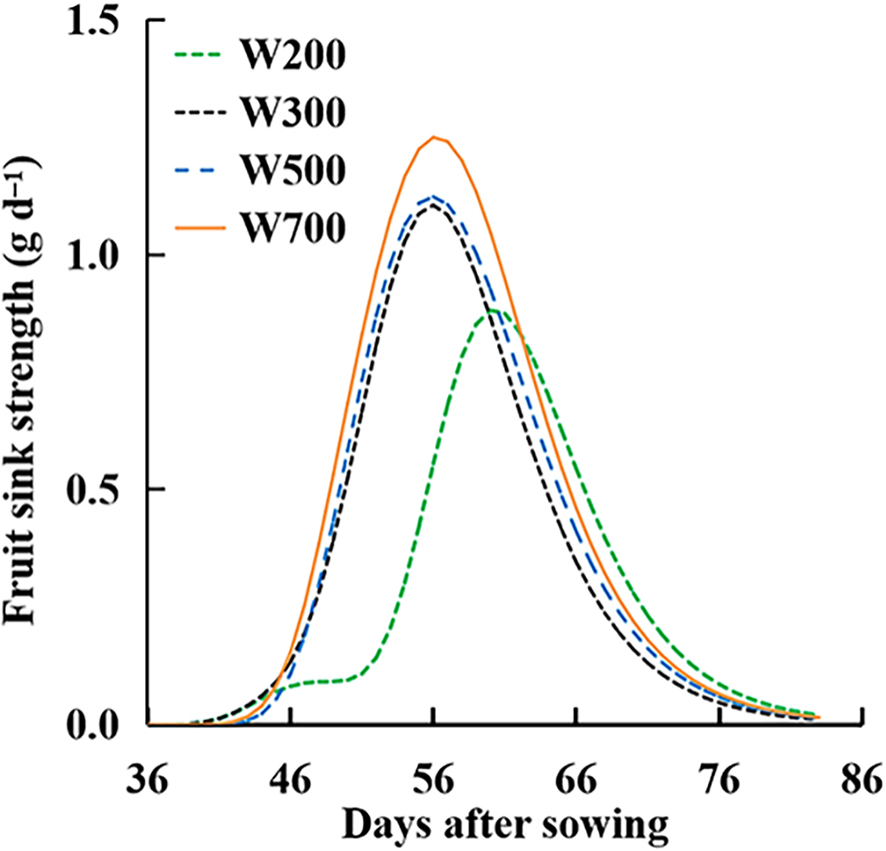
Effect of PPFD on fruit sink strength (*S*_fruit-sink_) over time in ‘Micro-Tom’ with standard fruit load (no fruit pruned).

The *S*_fruit-sink_ in all treatments showed a rising to declining trend over time (**Figure 8**). The peaks of the *S*_fruit-sink_ increased with an increase in PPFD. Until 60 DAS, *S*_fruit-sink_ decreased with the decrease in PPFD and was the lowest in the W200 treatment among all treatments. The *S*_fruit-sink_ in the W200 treatment was the highest, from 63 to 82 DAS.

## 4 Discussion

### 4.1 High PPFD decreases FBRUE by reducing RUE and *F*_fruits_

High PPFD (500 and 700 µmol m^−2^ s^−1^) decreased FBRUE by decreasing both RUE and *F*_fruits_ (**Figure 4**), which was consistent with our hypothesis. In addition, PPFD affected RUE more than *F*_fruits_. The influence of PPFD on *F*_fruits_ increased with an increase in PPFD. Until 56 DAS, RUE had a greater influence on FBRUE than *F*_fruits_ because there was no significant difference in *F*_fruits_ among all treatments (**Figure 5B**). From 56 DAS onwards, the impact of PPFD on *F*_fruits_ had a greater influence on FBRUE (**Figure 5**). Previous studies reported that the FBRUE of tomatoes cultivated in the same controlled environment agriculture systems at harvest was 0.2−0.36 g mol^−1^ (Wheeler et al., 2008; Goto, 2011; Li et al., 2019). In the present study, even the lowest FBRUE at harvest in W700 was 0.38 g mol^−1^ (**Figure 5A**; **Supplementary Table S2**), which was higher than others. This shows that the environmental control and variety selection used in the present study improved the FBRUE of tomatoes. We also verified that optimizing PPFD can improve FBRUE in dwarf tomatoes by improving RUE and *F*_fruits_. This was the first quantitative analysis of the impact of PPFD on FBRUE in dwarf tomatoes in a PFAL.

### 4.2 PPFD affects RUE by affecting leaf optical properties and photosynthesis

Light is one of the limiting resources in natural conditions, and plants grown under low PPFD conditions are required to adapt to capture light effectively (Lee and Graham, 1986). Conversely, leaves grown under low PPFD conditions have thicker cuticles and higher SLA and chlorophyll concentrations than those grown under high PPFD conditions (Araus and Hogan, 1994). Under high PPFD, leaves had higher reflectance and lower transmittance and absorptance than those under low PPFD (**Table 2**). Low absorptance under high PPFD could cause the integrated PPFDs received by the plant (*I*_PPFD_) to be overvalued and RUE to be undervalued.

In addition, plants are exposed to excessive amounts of light over a long period, producing large amounts of reactive oxygen species, superseding the antioxidant system, and resulting in irreversible photooxidative damage to chloroplasts and cells, thus preventing photosynthesis (Karpinski et al., 1997). A PPFD of 700 µmol m^−2^ s^−1^ might have been too high to decrease the *ϕ* (**Figures 2E, F**) and the photosynthetic capacity of the leaves (**Figure 2I**). This decrease became more significant over time (**Figure 2**). Higher PPFD led to lower *ϕ* at PPFDs of 200, 300, 500, and 800 µmol m^−2^ s^−1^ at 64 DAS (**Figures 2E, F**), which was the main reason high PPFD led to low RUE (**Figure 3**).

However, PPFD of 700 µmol m^−2^ s^−1^ did not inhibit biomass production until 82 DAS (**Table 1**). ‘Micro-Tom’ is known to grow, set, and ripen fruit even at extremely low light levels (PPFD: 100 µmol m^−2^ s^−1^; DLI: 5.76 mol m^−2^ day^−1^) (Frantz et al., 2000). However, few studies have reported whether a high PPFD can inhibit biomass production in ‘Micro-Tom’. The monthly averaged DLI in greenhouses rarely exceeded 30 mol m^−2^ day^−1^ (Higashide et al., 2014; Kaiser et al., 2019; Jin et al., 2022; Zhao et al., 2022). In addition, the most common DLIs in growth chambers are between 10 and 30 mol m^−2^ day^−1^ (Poorter et al., 2016). In this study, the DLIs of W200, W300, W500, and W700 were 11.52, 17.28, 28.80, and 40.32 mol m^−2^ day^−1^, respectively. Therefore, a DLI of 40.32 mol m^−2^ day^−1^ is high, even for general tomato cultivars. The light response of the whole canopy is different from the top single leaf, showing higher or no light-saturated points in extreme cases. Therefore, the PPFD of 700 µmol m^−2^ s^−1^ decreased the *ϕ* and *P*_max_ (**Figure 2**) of the top single leaf but did not inhibit biomass production of the whole canopy (**Table 1**). It is necessary to determine the direct relationship between the photosynthetic light response of the whole canopy and RUE in the future.

The growth stage can affect RUE too. The RUEs in W300, W500, and W700 at the vegetative growth stage were 1.15, 1.14, and 0.94 g mol^−1^, respectively (Ke et al., 2021). However, the RUEs in W300, W500, and W700 at the reproductive growth stage were 1.04, 0.85, and 0.78 g mol^−1^, respectively. The RUE during the reproductive growth stage was lower than that during the vegetative growth stage, even in the same cultivation environment. One reason might be that the leaf age at the vegetative growth stage was younger than at the reproductive growth stage. As a determinate tomato, ‘Micro-Tom’ plants stop shoot production on the main stem once flowering. The top leaves on the stem became older because no new leaves appeared on the main stem. In addition, the Pn decreased over time at the same PPFD (**Figures 2A–C**). Another reason is that the fruit was set on the top canopy, and the fruits were absorbed by the fruits. The gross photosynthetic rate per green fruit surface area is only 15–30% of the rate per leaf area (Czarnowski and Starzecki, 1990). Therefore, RUE decreased with the growth of fruit set in the canopy.

The RUE in W300 (1.04 g mol^−1^) was the highest (**Figure 3**). Therefore, 300 µmol m^−2^ s^−1^ PPFD was recommended for ‘Micro-Tom’ cultivation at the reproductive growth stage to improve RUE. Furthermore, Ke et al. (2021) reported that 300 µmol m^−2^ s^−1^ PPFD was proposed for ‘Micro-Tom’ cultivation during the vegetative growth stage to enhance the RUE. Therefore, 300 µmol m^−2^ s^−1^ PPFD can be applied to ‘Micro-Tom’ cultivation during vegetative and reproductive growth stages to enhance RUE.

### 4.3 PPFD affects *F*_fruits_ that associated with source strength and fruit sink strength

The *F*_fruits_ increased from 36 to 64 DAS and remained stable, ranging from 0.49 to 0.60 at harvest. Generally, the *F*_fruits_ (not including root dry mass) of year-round greenhouse indeterminate tomatoes was 69–72% (Cockshull et al., 1992; De Koning, 1993). For field-grown semi-determinate tomatoes, *F*_fruits_ (excluding root dry mass) was 53–71%, with an average of 58% (Scholberg et al., 2000), and for processing tomatoes, it ranged from 57% to 67% (Hewitt and Marrush, 1986; Cavero et al., 1998). In the present study, the fraction of dry mass partitioned to the root was approximately 10% (**Supplementary Figure S5**); therefore, the *F*_fruits_ (not including root dry mass) in the present study was 56–65%, which is similar to the values reported in previous studies.

In addition, fruit dry weight increased by 16.1% and 35.7% when PPFD at the top of plants increased by 157.8% (W500) and 261.0% (W700), respectively, from 200 µmol m^−2^ s^−1^ (**Figures 4B, C**). In practice, the ‘1% rule’ is often used to estimate the impact of light on the production, stating that an increase in light by 1% will result in an increase in production by 1%. For tomatoes, this value varies between 0.7% and 1% (Marcelis et al., 2006). However, ‘Micro-Tom’ is a determinate tomato cultivar that is different. The number of fruits on the main stem is limited. Therefore, the fruit sink’s strength is limited. This might be why the *F*_fruits_ decreased with an increase in PPFD in the present study, while the *F*_fruits_ of indeterminate tomatoes increased with an increase in PPFD (Yan et al., 2018).

The *W* (**Figure 6A**) and the *S*_source_ (**Figure 6B**) increased with an increase in PPFD. However, *F*_fruits_ did not increase with an increase in PPFD from 56 DAS. This means that the dry mass-produced was transferred more to leaves and roots than to the target organ fruits at high PPFDs (**Supplementary Figure S5**). The main reason was that the *S*_fruit-sink_ (**Figure 8**) decreased from 56 DAS at high PPFDs. Therefore, high *S*_source_ and low *S*_fruit-sink_ led to low *F*_fruits_ at high PPFD during the late reproductive growth stage. Two factors can directly affect the *S*_fruit-sink_: the sink strength of each single fruit (potential growth rate of individual fruit (IGR_fruit_)) and the number of fruits. PPFD did not affect the potential growth rate of individual fruits (**Figure 7D**) in ‘Micro-Tom’, which was similar to a previous study (Marcelis, 1996). However, the number of fruits and the peak of *S*_fruit-sink_ increased with an increase in PPFD (**Figure 8**). This was the first discussion of how PPFD affects source and fruit sink strength, clarifying how PPFD affects dry matter distribution in dwarf tomatoes under LED light.

High PPFD is necessary at the early reproductive growth stage to induce flower bud differentiation and improve the number of flowers (Samach and Lotan, 2007) and fruit sink and yield. Generally, starch, particularly in the columella, placenta, and inner and radial pericarps (Schaffer and Petreikov, 1997), is filled in the early phase of fruit expansion and peaks around 10–25 DAA (Bertin et al., 2009). In indeterminate tomatoes, the *S*_fruit-sink_ was initially low, soon increased to a plateau, and remained constant until 100 days after planting (Li et al., 2015). However, high PPFD and *S*_source_ might not be necessary for ‘Micro-Tom’ at the late reproductive growth stage (from 64 DAS), when *F*_fruits_ (**Figure 5B**) was stable and *S*_fruit-sink_ (**Figure 8**) was low. Because there were no new fruits on the main stem at the late reproductive growth stage, dynamic PPFD management, high PPFD before 64 DAS, and low PPFD from 64 DAS might be suitable for improving FBRUE and yield in ‘Micro-Tom’ at the reproductive growth stage.

## 5 Conclusions

Our study showed that FBRUE increased slightly with an increase in PPFD from 200 to 300 µmol m^−2^ s^−1^ and decreased because of the decreases in RUE and *F*_fruits_ when PPFD increased from 300 to 700 µmol m^−2^ s^−1^. From 300 to 700 µmol m^−2^ s^−1^ PPFD, higher PPFD led to lower RUE because of lower *ϕ* and *P*_max_. In addition, *S*_source_ and *S*_fruit-sink_ increased with an increase in PPFD. PPFD did not affect the potential growth rate of individual fruits but the number of fruits. At the late reproductive growth stage, high *S*_source_ and low *S*_fruit-sink_ led to low *F*_fruits_ at 700 µmol m^−2^ s^−1^ PPFD. In summary, 300 µmol m^−2^ s^−1^ PPFD is recommended for ‘Micro-Tom’ cultivation to improve FBRUE and RUE at the reproductive growth stage. Furthermore, dynamic PPFD management based on the source-sink relationship might be suitable for improving FBRUE and yield in ‘Micro-Tom’ during the reproductive growth stage. The results of this study would be helpful in efficient tomato production in PFALs and may help elucidate the effects of PPFD on FBRUE, source strength, and fruit sink strength of dwarf tomatoes under LED light. In addition, the light quality is also a key consideration for improving RUE and FBRUE. Further research is necessary for detecting the optimal combination of PPFD and light quality to enhance RUE and FBRUE in dwarf tomatoes.

## Data availability statement

The original contributions presented in the study are included in the article/**Supplementary Material**. Further inquiries can be directed to the corresponding author.

## Author contributions

Conceptualization, methodology, and design of the experiment: XK and EG. Performed the experiment, collected the samples for analysis, parameter measurement, and statistical analysis of data: XK. Writing—original draft preparation: XK. Writing—review and editing: EG, HY, and SH. Supervision and funding acquisition: EG. All authors have read and agreed to the published version of the manuscript.
