# Supplementary material for: Photosynthetic photon flux density affects fruit biomass radiation-use efficiency of dwarf tomatoes under LED light at the reproductive growth stage
Source: Front Plant Sci. 2023 Feb 27;14:1076423. doi: 10.3389/fpls.2023.1076423 (PMC10009779; doi:10.3389/fpls.2023.1076423)
Supplement: Supplementary file 1 [file DataSheet_1.docx]

Supplementary Material

## Supplementary Figures and Tables

## Supplementary Figures


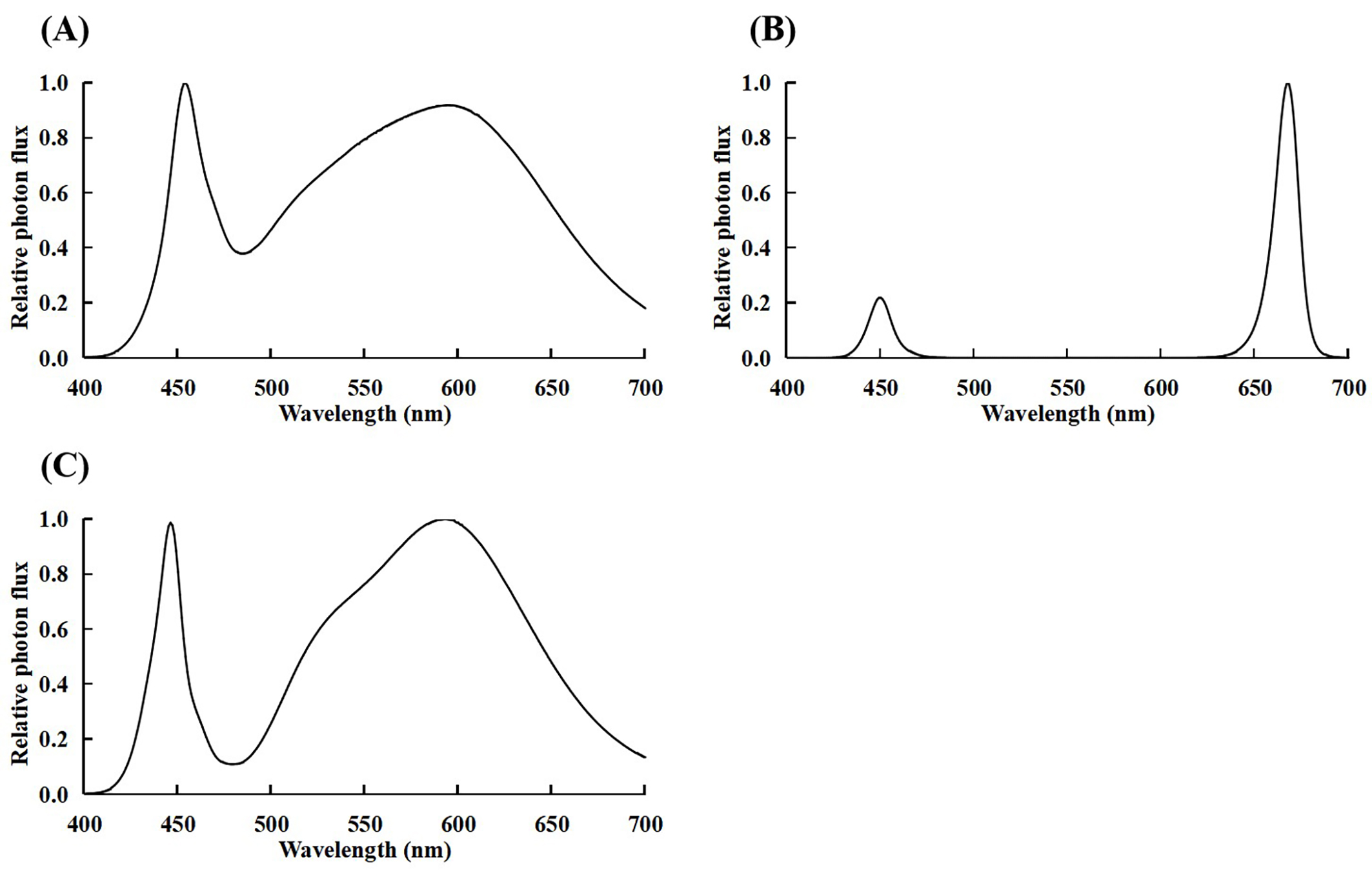
**Supplementary Figure 1. Spectral photon flux distributions of (A) white lamps (LDL40S-N19/21) until 24 days after sowing (DAS), (B) red and blue (red:blue = 9:1) LED lamps (CIVILIGHT) from 24–35 DAS, and (C) white LED lamps (customized lamp) after 35 DAS.** The maximum value of photon flux was converted to 1.0.

##
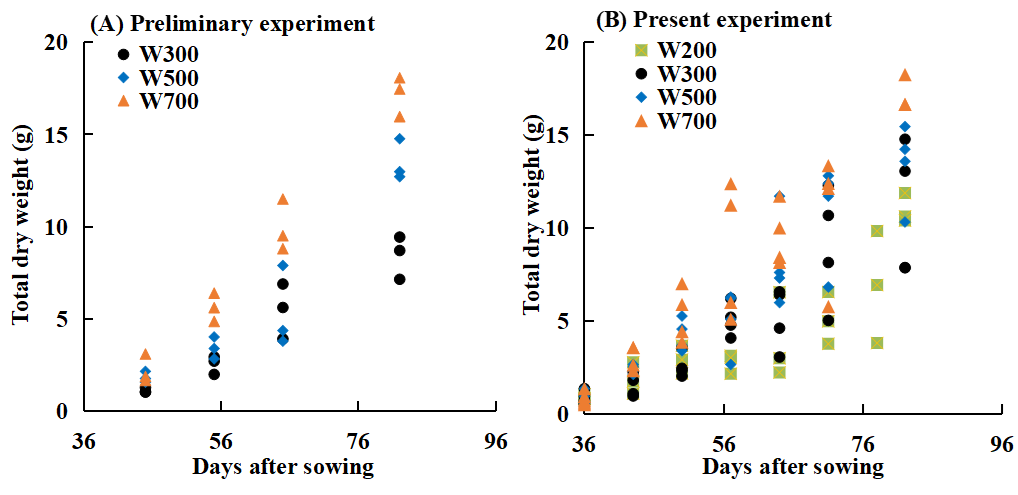
Supplementary Figure 2. The total dry weight of a ‘Micro-Tom’ plant from 36 to 84 days after sowing (DAS) in different photosynthetic photon flux density (PPFD) treatments in a preliminary experiment (A) and present experiment (B).


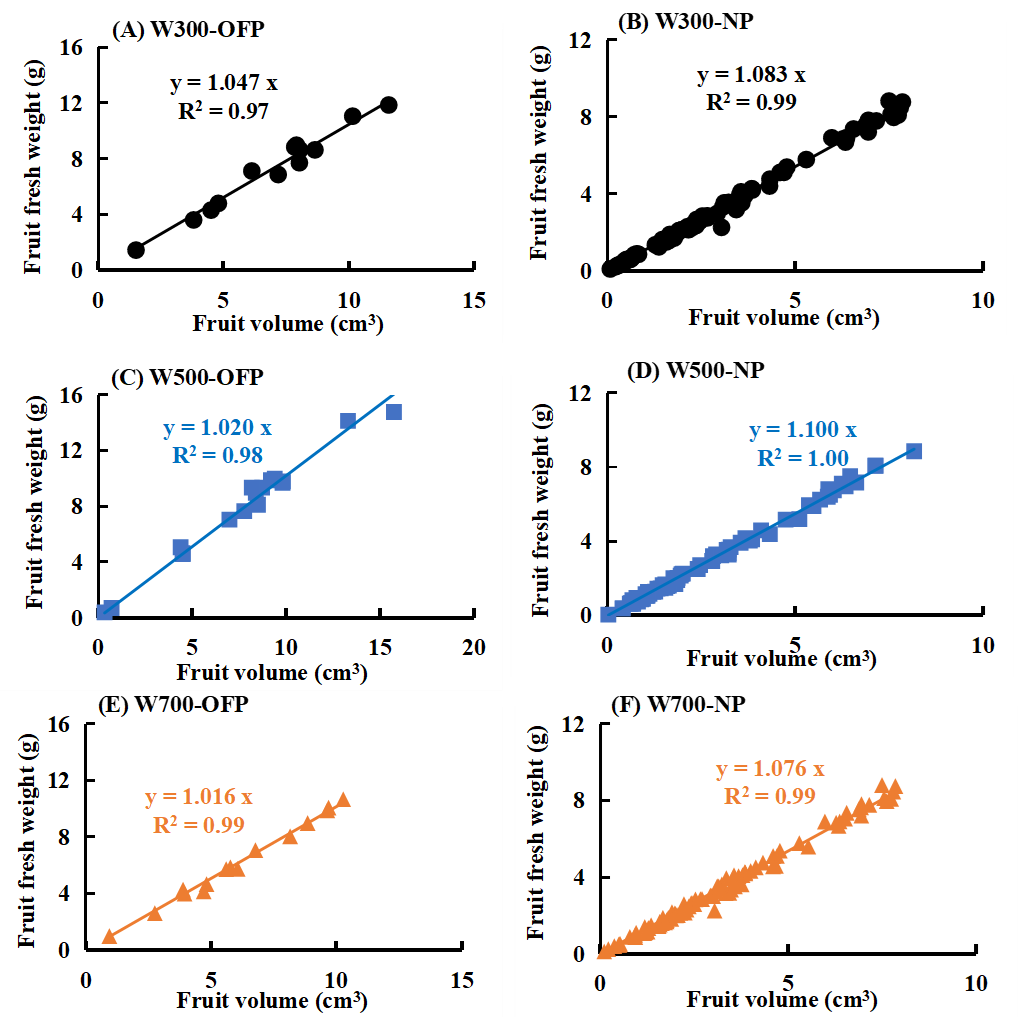


**Supplementary Figure 3. The relationships between individual fruit fresh weight and volume of one-fruit plants (A) and plants without fruit pruning (B) in W300, of one-fruit plants (C) and plants without fruit pruning (D) in W500, and one-fruit plants (E) and plants without fruit pruning (F) in W700.** There were 15–16 fruits of one-fruit plants, and 77–104 fruits of plants without fruit pruning sampled in each PPFD treatment.


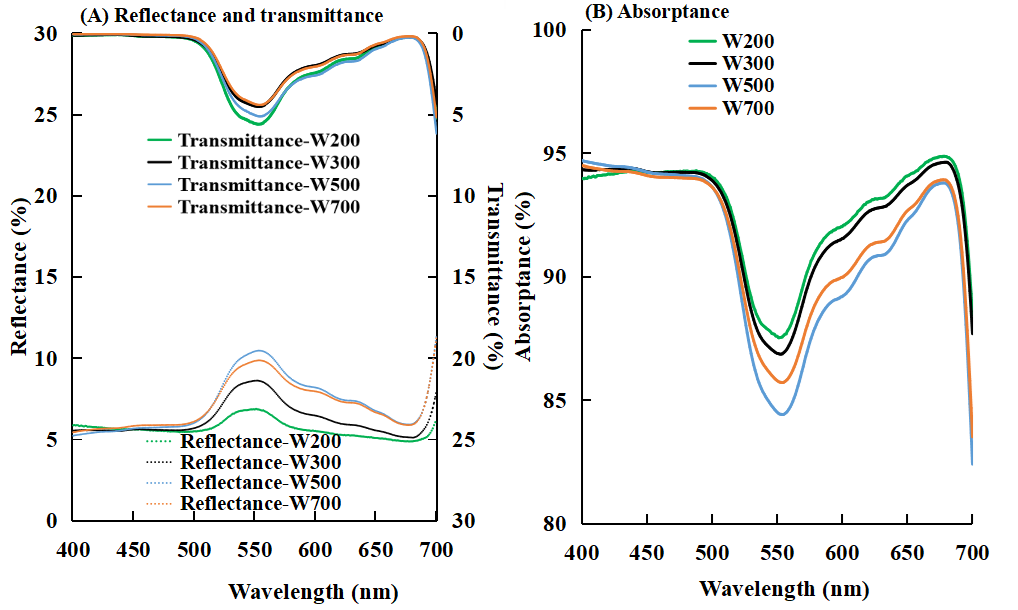


**Supplementary Figure 4. Effects of PPFD on the spectra of reflectance and transmittance (A) and absorptance (B) of leaves in ‘Micro-Tom’ 82 DAS.** The range of measured light spectrum was 400–700 nm. W200, W300, W500, and W700 denote 200, 300, 500, and 700 µmol m^−2^ s^−1^ PPFD, respectively. Each value represents the average of the values of four NPs.


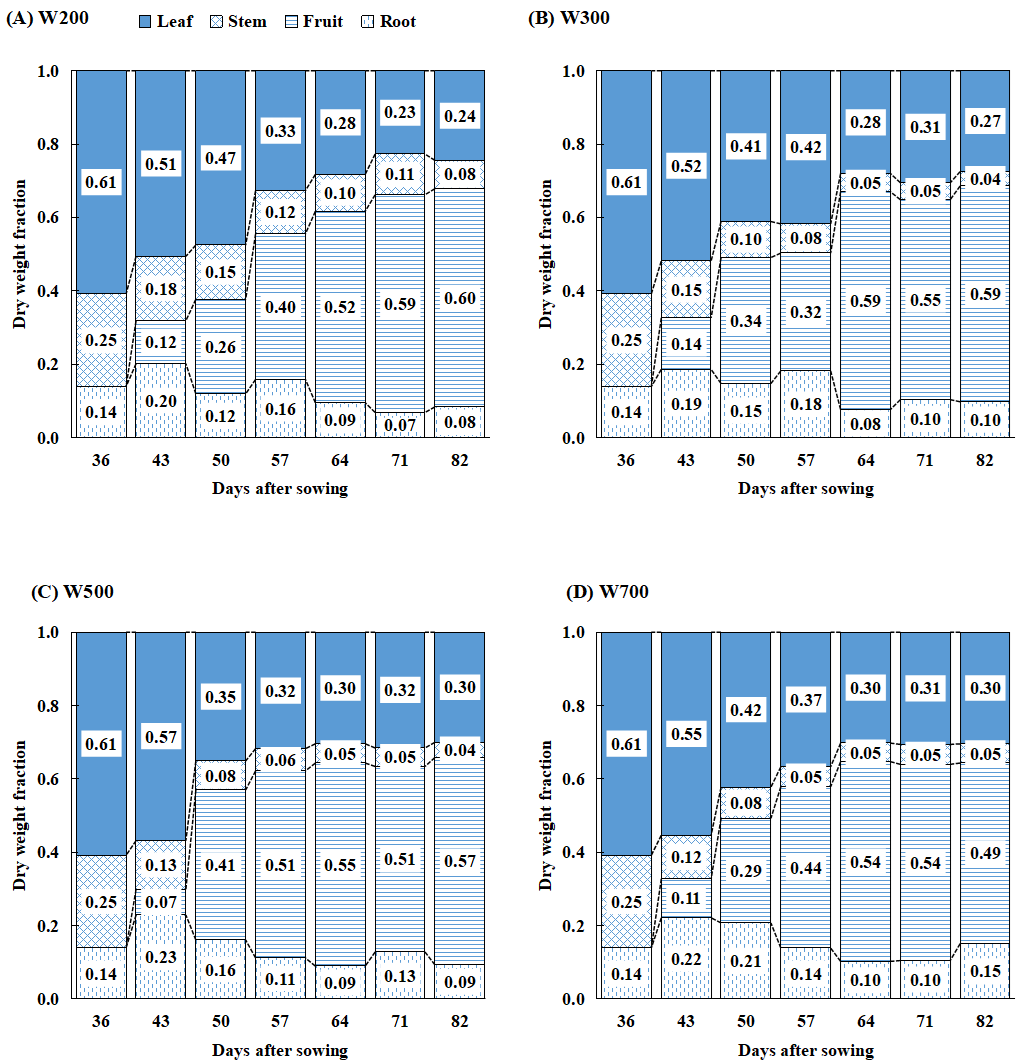


**Supplementary Figure 5. Fractions of dry mass partitioned to each organ in W200 (A), W300 (B), W500 (C), and W700 (D) treatments in ‘Micro-Tom’ 36, 43, 50, 57, 64, 71, and 82 days after sowing (DAS).** Each value represents the mean of three or four values. All sampled plants are NPs.

**Supplementary Figure 6. The relation between fruit age and dry matter content of one-fruit plants in W300, W500, and W700 treatments.** There were 11–15 fruits sampled in each PPFD treatment. Curves represent the 4th-degree (or 3rd-degree) polynomial function used to fit the data in the three treatments (Equation 6). Goodness of fit of fitted curves are shown in the box. SSE, R^2^, adjusted R^2,^ and RMSE represent the sum of squares due to error, coefficient of determination, degree-of-freedom adjusted coefficient of determination, and root mean squared error, respectively.

## Supplementary Tables

**Supplementary Table 1. Goodness of fit of the fitted total dry weight curves related to Equation 4 in the present study.** The SSE, R^2^, adjusted R^2^, and RMSE represent the sum of squares due to error, coefficient of determination, degree-of-freedom adjusted coefficient of determination, and root mean squared error, respectively.

| Treatment | SSE | R^2^ | Adjusted R^2^ | RMSE |
| --- | --- | --- | --- | --- |
| W200 | 2.1 | 0.97 | 0.96 | 0.64 |
| W300 | 2.6 | 0.97 | 0.96 | 0.72 |
| W500 | 3.9 | 0.96 | 0.94 | 0.99 |
| W700 | 5.1 | 0.96 | 0.95 | 1.13 |

**Supplementary Table 2. Effects of PPFD on parameters of fruit biomass radiation-use efficiency (FBRUE) component analyses.** The results are shown in Fig. 6. Abbreviations within the table are as follows: FBRUE, fruit biomass radiation-use efficiency, g mol^−1^; RUE, radiation use efficiency, g mol^−1^; F_fruits_, fraction of dry mass partitioned to fruits, g g^–1^; I_PPFD_, integrated PPFD received by the plant until 82 DAS, mol; W, total dry weight, g; W_fruit_, fruit dry weight, g; PPFD_T_, difference between the PPFDs at the top and bottom of the plant (mol m^− 2^ s^− 1^); and average PLA, average projected leaf area, m^2^. All sampled plants were treated with NPs. All values except FBRUE, RUE, and I_PPFD_ represent the mean ± standard error. Different letters indicate significant differences at *p* < 0.05 (*n* = 3−4) among PPFD treatments with Tukey−Kramer’s test.

| **Treatment** | **FBRUE**  **(g mol^−1^)** | **RUE**  **(g mol^−1^)** | **F_fruits_**  **(g g^–1^)** | **I_PPFD_**  **(mol)** | **W**  **(g)** | **W_fruit_**  **(g)** | **PPFD_T_**  **(mol m^−2^ s^−1^)** | **Average PLA**  **(m^2^)** |
| --- | --- | --- | --- | --- | --- | --- | --- | --- |
| W200 | 0.59 | 1.00 | 0.59 ± 0.06 a | 11.0 | 10.97 ± 0.46 b | 6.53 ± 0.70 c | 192.2 ± 0.5 d | 180.7 ± 11.2 a |
| W300 | 0.61 | 1.04 | 0.59 ± 0.03 a | 10.5 | 10.90 ± 1.78 b | 6.41 ± 1.05 c | 297.0 ± 0.6 c | 119.5 ± 3.4 b |
| W500 | 0.48 | 0.85 | 0.57 ± 0.02 a | 15.8 | 13.41 ± 1.10 ab | 7.58 ± 0.56 b | 495.4 ± 1.13 b | 117.2 ± 3.7 b |
| W700 | 0.38 | 0.78 | 0.49 ± 0.03 b | 23.1 | 18.00 ± 0.89 a | 8.86 ± 0.83 a | 693.6 ± 1.7 a | 117.2 ± 3.6 b |

**Supplementary Table 3. Effects of fruit pruning on** **volume, fresh and dry weights, and dry matter content 42 days after anthesis (DAA) in W300, W500, and W700 treatments.** Each value represents the mean ± standard error.

| **Treatment** | **PPFD**  **(μmol m^–2^ s^–1^)** | **Pruning treatment** | **Fruit age (DAA)** | **Fruit volume**  **(cm^3^)** | **Single fruit fresh weight (g/fruit)** | **Single fruit dry weight (g/fruit)** |
| --- | --- | --- | --- | --- | --- | --- |
| W300 | 300 | One-fruit plants | 42 | 8.76 ± 0.95 | 9.54 ± 0.85 | 0.98 ± 0.11 |
|  |  | Plants with one fruit per truss |  | 9.78 ± 0.36 | 10.59 ± 0.39 | 1.00 ± 0.04 |
| W500 | 500 | One-fruit plants | 42 | 9.14 ± 0.78 | 9.59 ± 0.86 | 1.12 ± 0.05 |
|  |  | Plants with one fruit per truss |  | 7.93 ± 1.38 | 8.63 ± 1.60 | 1.00 ± 0.14 |
| W700 | 700 | One-fruit plants | 42 | 8.55 ± 0.83 | 8.64 ± 0.97 | 1.30 ± 0.13 |
|  |  | Plants with one fruit per truss |  | 8.72 ± 1.21 | 8.59 ± 1.37 | 1.09 ± 0.17 |

**Supplementary Table 4. List of abbreviations and symbols.**

| **Abbreviation or symbol** | **Full name or description** | **Unit** |
| --- | --- | --- |
| DAS | Days after sowing | days |
| DLI | Daily light integral | mol m^–2^ d^–1^ |
| EC | Electrical conductivity | dS m^–1^ |
| FBRUE | Fruit biomass radiation-use efficiency | g mol^−1^ |
| F_fruits_ | Fraction of dry mass portioned to fruits | - |
| *IDMC_fruit_(x)* | Dry matter content of individual fruits at *x* days after anthesis (DAA) | - |
| *IGR_fruit_* | Growth rate of individual fruit | g d^–1^ |
| I_PPFD_ | Integrated PPFD | mol |
| *IW_max_* | Maximum dry weight of individual fruit | g |
| *k* | Growth rate coefficient | **-** |
| LA | Leaf area | cm^2^ |
| PFAL | Plant factory with artificial light | **-** |
| *PLA(t)* | Projected leaf area of the plant on day *t* | m^2^ |
| P_max_ | Maximum net photosynthetic rate | µmol m^−2^ s^−1^ |
| Pn | Net photosynthetic rate | µmol m^−2^ s^−1^ |
| PPFD | Photosynthetic photon flux density | µmol m^−2^ s^−1^ |
| *PPFD(t)* | PPFD at the bottom of the canopy on day *t* | mol m^–2^ s^–1^ |
| *PPFD_T_* | PPFD at the top of the canopy | mol m^–2^ s^–1^ |
| RUE | Radiation-use efficiency | g mol^−1^ |
| S_fruit-sink_ | Fruit sink strength | g d^−1^ |
| SLA | Specific leaf area | cm^2^ g^−1^ |
| S_source_ | Source strength | g d^−1^ |
| W | Total dry weight | g |
| *W_fruits_* | Fruit dry weight | g |
| *x* | Fruit age | DAA |
| *x_m_* | Fruit age at the maximum growth rate | DAA |
| ϕ | Photosynthetic quantum yield | mmol CO_2_ / mol photon |
